# A Moderated e-Forum for Adults With Cardiovascular Disease: Usability Study

**DOI:** 10.2196/humanfactors.8820

**Published:** 2018-05-18

**Authors:** Rika Tanaka, Anita Banerjee, Jelena Surikova, Jacqueline Tracey, Ada Payne, Heather Ross, Robert Nolan

**Affiliations:** ^1^ Cardiac eHealth and Behavioural Cardiology Research Unit Peter Munk Cardiac Centre University Health Network Toronto, ON Canada; ^2^ Models of Care Clinical Programs and Quality Initiatives Cancer Care Ontario Toronto, ON Canada; ^3^ Faculty of Medicine University of Toronto Toronto, ON Canada; ^4^ Ted Rogers Centre of Excellence in Heart Function Peter Munk Cardiac Centre University Health Network Toronto, ON Canada

**Keywords:** support groups, cardiovascular disease, qualitative research

## Abstract

**Background:**

Self-care behaviors are commonly prescribed to manage both cardiovascular disease and hypertension to reduce modifiable risk factors and improve quality of life. Nevertheless, long-term adherence to self-care recommendations for cardiac patients has been problematic. In cardiac patients, moderated online forums have been found to be particularly useful in supporting maintenance of heart-healthy diet and fewer hospital visits. As such, we developed the e-Forum, a Web-based moderated forum designed to promote continued user engagement and long-term self-care adherence.

**Objective:**

The objective of this study was to assess the usability of the user interface for the newly designed e-Forum. In addition to overall user satisfaction, we obtained feedback from our target users on the key features of this newly developed interface.

**Methods:**

An iterative design tested the usability of the e-Forum. On the basis of the user feedback, adjustments were made to the design of our e-Forum, and these changes were then tested in the succeeding group. Participants were recruited from the Heart Function Clinic at the Peter Munk Cardiac Center, University Health Network. After consenting to participate in our study, patients were asked to complete a set of goal-oriented tasks and a feedback interview for the e-Forum. A content analysis of the transcripts from the set of goal-oriented tasks and feedback interviews identified several themes, including general feedback and comments regarding 3 key areas of the e-Forum: layout, navigation, and content.

**Results:**

Overall, 13 cardiac patients (aged 32-81 years) participated in 3 rounds of testing. Participants across all 3 rounds were highly satisfied with our e-Forum and indicated that they would find such a forum useful in managing their health. Expressions of overall satisfaction with the e-Forum and positive comments regarding layout increased between the initial and the final round. As improvements were made to the e-Forum based on participant feedback, potential barriers, negative comments related to the content, and the number of navigation errors decreased between rounds 1 and 3.

**Conclusions:**

We found evidence to support the usability of the user interface for our e-Forum. These results indicate that the e-Forum will likely be a successful tool to support an online community of cardiac patients in their efforts to sustain long-term lifestyle behavior change.

## Introduction

### Overview

According to the American Heart Association, cardiovascular disease (CVD) accounted for approximately 1 in every 3 deaths in the United States in 2013 [1]. Self-care behaviors (eg, maintaining a healthy diet, regular exercise, and medication adherence) are recommended to manage both CVD and hypertension to reduce modifiable risk factors and improve quality of life [2]. Nevertheless, long-term adherence to self-care recommendations for cardiac patients has been problematic [3]. In an effort to reduce risk for CVD and improve quality of life for patients, our research team developed a Web-based lifestyle counseling platform for cardiac patients (eg, those diagnosed with hypertension or heart failure, HF) to promote adherence to self-care recommendations [4-9].

On the basis of evidence from our program of research, our team created the Canadian e-Platform to Promote Behavioral Self-Management in Chronic Heart Failure (CHF-CePPORT; ClinicalTrials.gov: NCT01864369) [10]. Although CHF-CePPORT provides a 12-month comprehensive e-counseling program for self-care behavior change in patients with HF, long-term adherence to Web-based lifestyle counseling programs can be difficult to sustain. For example, dropout rates in Web-based interventions can range up to 62%, and failure to participate in the e-based interventions is 28% over 9 months [11]. These findings indicate that such programs may benefit from supplementary features that facilitate long-term patient engagement and adherence. To address this issue, we developed the e-Forum to supplement CHF-CePPORT by supporting the establishment of an online community that aims to promote continued user engagement and long-term self-care adherence. Our aim was to tailor the design and functional features of the e-Forum to meet the needs of patients with cardiovascular conditions such as HF, who are likely to be older and to present with lower computer literacy. In keeping with guidelines suggested from previous research [12-14], this study assessed the usability of this e-Forum to determine whether cardiac patients could use this program as intended.

### Web-Based Moderated Forums

The use of online social networks is an important method for facilitating information sharing as well as providing and receiving support among patients and health care professionals [15,16]. Online communities offer patients access to both emotional support and information about disease management that are not always available or easily accessible [17]. Online moderated forums are online communities that are monitored by professionals or trained peers who (1) facilitate user engagement in the online forum, (2) ensure the accuracy of information discussed by users, and (3) check for safety and appropriateness of posted messages (eg, monitoring for language suggesting self-harm or aggressive or offensive language). Patients demonstrate a preference for this type of intervention over and above conventional e-pages that only present information [18,19]. Such forums have been found to help a diverse array of patients, including those suffering from obesity [17] and ovarian cancer [20]; they offer users a resource to manage the complexities of their illnesses by promoting and supporting healthy self-care strategies [21]. In cardiac patients, such online communities have been found to be particularly useful in supporting maintenance of heart-healthy diet and fewer visits to the hospital [22].

**Figure 1:**
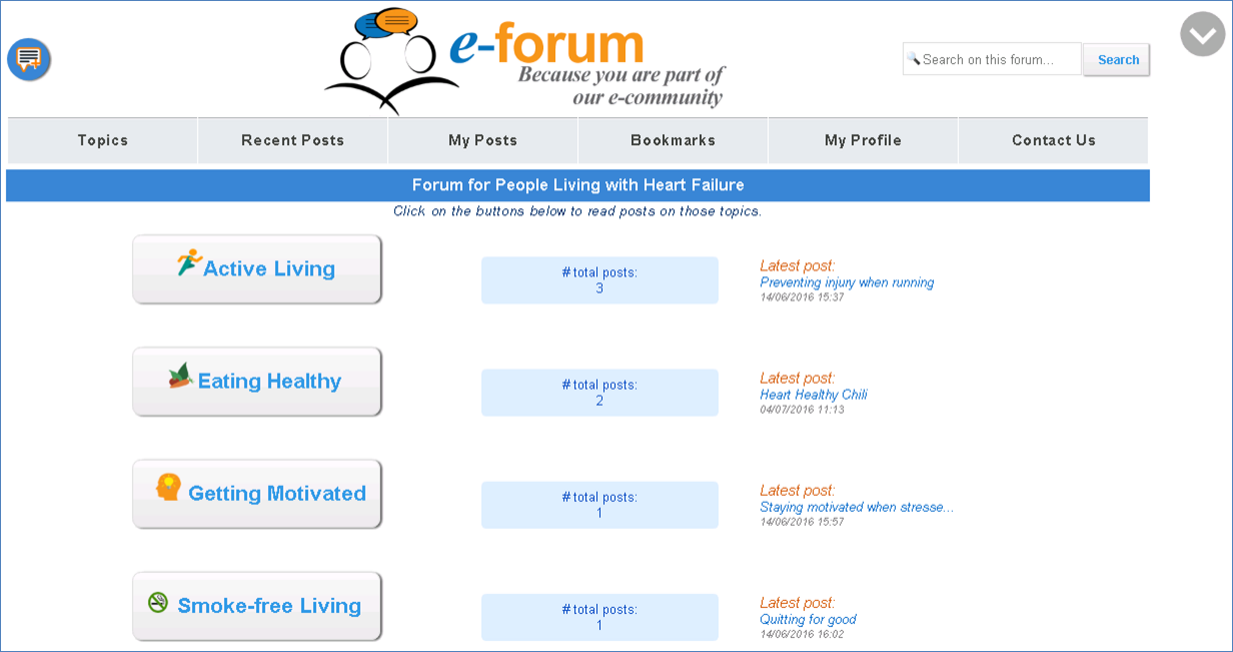
Final version of e-Forum home page. On the basis of user feedback, the final version of the e-Forum home page included a button on the upper left corner that allows users to add a post to the forum without first accessing a topic. It also included a search function as well as a scroll button on the upper right corner.

**Figure 2:**
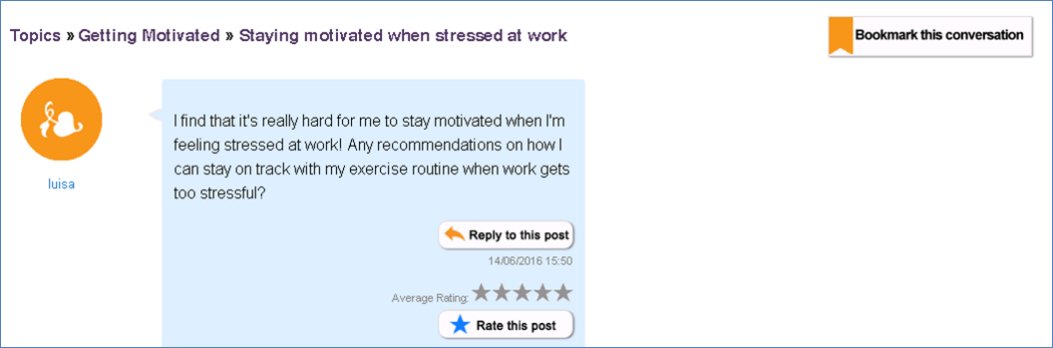
Final version of post thread. On the basis of user feedback, the final version of the post thread included a breadcrumb feature on the upper left corner that allows users to see what topic and thread they are reading. Buttons on this page were also redesigned to consistently include both icons and verbal descriptions of the button functions.

**Figure 3:**
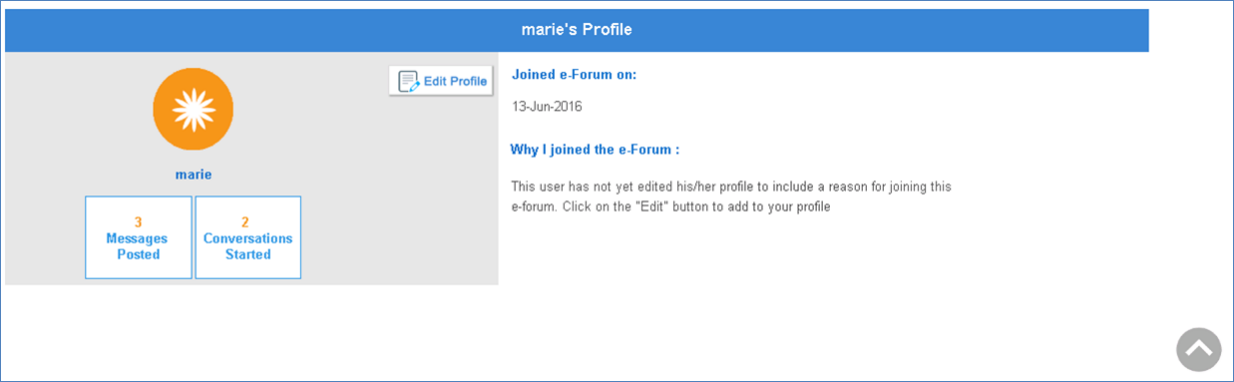
Final version of My Profile page. The final version of the “My Profile” page reflected user feedback in that it guided users to edit their profile by including both an icon and a verbal description of this button’s function.

We designed our e-Forum to provide a reliable and accessible interface to foster an online community for patients enrolled in our CHF-CePPORT program. From a functional perspective, our e-Forum was developed to allow users to submit *posts*, including comments or questions regarding their efforts to begin or maintain therapeutic changes in self-care behaviors. The e-Forum was organized such that posts may be submitted under highlighted topics, including “Active Living,” “Eating Healthy,” “Smoke-free Living,” and “Getting Motivated” (see Figures 1-3 to view the final version of the e-Forum). The e-Forum was designed to then send submitted posts to a moderator, who was trained to review posts for accuracy and appropriateness of content and patient safety before they were made accessible and viewable to the other members of the online community. In addition, the e-Forum was designed to allow members of our team to host live or taped presentations on select topics related to self-care adherence and quality of life. The original prototype of the e-Forum also featured large buttons, bright and inviting colors, and large font sizes to increase usability for our older target patient population.

### Usability Assessment

Although there is preliminary evidence that the use of online forums may be an effective mode of intervention to enhance education and therapeutic support for participants, it is unclear which features enable users to interact with such forums more effectively [23-25]. Therefore, we undertook a usability study to assess our high-quality, user-centered interface designed to maximize the engagement with the e-Forum [26]. Specifically, our usability study was conducted to determine whether the target users (ie, cardiac patients) could use the e-Forum as intended. Usability studies have been found to improve the design of several other Web-based programs. For example, Stinson et al conducted a usability study to improve their Web-based self-management program for adolescents with arthritis and their parents [27]. In the first of 2 rounds of usability testing, adolescents with arthritis and their parents reported that the labels used in the medication home page were ambiguous, resulting in navigation difficulties in that portion of the program. On the basis of this feedback, the team revised the labeling, and this issue was not reported in the second round of the usability testing. A usability study of a Web-based self-management program for patients diagnosed with chronic obstructive pulmonary disease also found this type of assessment to be helpful in improving the design of their program [13]. The CHF-CePPORT program prototype also underwent a usability study [14]. During this study, navigation issues were identified and resolved before its launch as part of a randomized controlled trial [14]. Together, these studies suggest that users can provide practical feedback to help identify problems with functionalities that may have otherwise been overlooked.

### Objective

The objective of this study was to assess the usability of the user interface for our newly designed e-Forum. To achieve this goal, we obtained feedback from our target users (eg, cardiac patients) on key features including general feedback, overall user satisfaction, layout, navigation, and content of the e-Forum.

## Methods

### Study Design

An iterative design [26,28] examined the usability of the e-Forum, such that multiple groups of participants were asked to navigate the e-Forum. On the basis of analysis of feedback from each round, adjustments were made to the e-Forum; these changes were then assessed for usability with the succeeding group of new participants.

### Participant Recruitment

Because the e-Forum aims to foster heart-healthy lifestyle changes that are applicable to all cardiac patients, including HF patients, we wanted to ensure that it was user-friendly to the wider, heterogeneous cardiac population. Thus, we recruited subjects from the Heart Function Clinic at the Peter Munk Cardiac Center, University Health Network. Patients were eligible to participate in this study if they were (1) male or female patients aged ≥18 years, (2) diagnosed with a CVD, including systolic HF with New York Heart Association Class I-III symptoms, and (3) fluent in English. To assess whether our e-Forum was easy to use for individuals with varying degrees of experience, we purposefully sampled an array of self-reported novice and advanced users of both computers and the internet. Individuals who did not use computers and the internet at all and were not willing to try these technologies were ineligible to participate in this study.

### Procedure

This study received approval from the Research Ethics Board at the University Health Network. During the study visit, each consented participant was asked to complete a set of goal-oriented tasks and a feedback interview on the e-Forum. All study visits were completed within 1.5 hours.

Goal-oriented tasks were the same across all study rounds and included logging onto the website, watching a tutorial video, and using different features of the e-Forum (eg, editing sample user profiles, submitting, bookmarking, and rating sample posts; Multimedia Appendix 1). Instructions for each goal-oriented task were read to participants, before asking them to “think-aloud” as they completed each task [29]. This commonly used protocol allowed us to assess the ongoing thought processes and difficulties experienced by the users while using the program [29]. To prevent disruption in the think-aloud protocol, no guidance or assistance was provided during task completion, unless requested by the participant [29]. All participants were able to successfully complete the think-aloud protocol.

After completing the set of goal-oriented tasks, a semistructured interview was used to ask participants about their overall experience with the e-Forum and to allow them to make suggestions for its improvement in layout (eg, font size, colors, and formatting), navigation (eg, ease of use), and content (eg, highlighted topics, and features/functionalities, including bookmarking and rating functions). All think-aloud sessions and feedback interviews were audio-taped using a digital audio recorder and then transcribed verbatim for analysis. Finally, all subjects completed a demographics form and a user satisfaction questionnaire. The items on the user satisfaction questionnaire were based on the usability characteristics, as described by Nielson [30], and included a 5-point Likert scale (1=“disagree very much”; 5=“agree very much”) asking participants to rate their level of satisfaction with different aspects of the e-Forum.

### Data Analysis

After each study visit, a research assistant transcribed the audiotape verbatim, and a second research assistant independently compared this transcription with the audiotape to verify its accuracy. A content analysis of the transcripts from the study sessions identified themes related to the overall satisfaction and the layout, navigation, and content of the e-Forum. QSR NVivo (QSR International, Victoria, Australia) was used to manage the transcript data. Concurrent data collection and analysis and constant comparison [31] facilitated probing for further insights to confirm themes that arose in subsequent interviews [32]. Transcripts were independently coded by RT and AB, and divergent codes were discussed and resolved. Once the coding process was complete, a frequency count tallied participants’ experiences in each theme [32]. Both quantitative frequency counts and qualitative interview excerpts were reported. Means, SDs, and percentages were calculated for data collected from the demographics and the satisfaction questionnaire forms.

## Results

### Participants

A total of 9 men and 4 women participated in this study over 3 rounds of data collection (n_round 1_=5, n_round 2_=5, and n_round 3_=3). Saturation of the narrative data was obtained with this sample. Participants’ age ranged from 32 to 81 years (mean=63.1, SD=13.8). The majority of participants were white (77%, 10/13), married (62%, 8/13), and had at least some postsecondary education (77%, 10/13). Six (46%, 6/13) participants were employed at the time of the study session. With regard to diagnosis, 7 participants (54%, 7/13) had been diagnosed with HF or cardiomyopathy, and 6 (46%, 6/13) had been diagnosed with other CVD, including cardiac amyloidosis, valvular heart disease, or ischemic heart disease (Table 1).

All 13 participants used the internet at home, with 12 accessing the internet via a computer and 1 via a mobile device. All participants reported at least being somewhat comfortable with computers and the internet. Nevertheless, there was variability in the degree to which participants used the computer/internet at home. Of the participants who had a computer at home, 50% (6/13) spent less than 5 hours per week on the computer, whereas the other half (6/13) spent more than 5 hours per week on the computer.

**Table 1:** Demographic characteristics of study participants.

Demographic variables		Round 1 (n=5), n (%)	Round 2 (n=5), n (%)	Round 3 (n=3), n (%)
**Age in years**				
	30-49	1 (20)	2 (40)	0 (0)
	50-69	3 (60)	1 (20)	2 (67)
	>70	1 (20)	2 (40)	1 (33)
**Gender**				
	Male	5 (100)	2 (40)	2 (67)
	Female	0 (0)	3 (60)	1 (33)
**Marital status**				
	Married/common-law	3 (60)	4 (80)	1 (33)
	Single/separated/divorced	2 (40)	1 (20)	2 (67)
**Highest education level**				
	High school	2 (40)	1 (20)	0 (0)
	Some college/college	1 (20	1 (20)	1 (33)
	Graduate/professional degree	2 (40)	3 (60)	2 (67)
**Employment status**				
	Full-time/part-time	2 (40)	2 (40)	2 (67)
	Retired/disability/leave of absence	3 (60)	3 (60)	1 (33)
**Ethnicity**				
	White	3 (60)	4 (80)	3 (100)
	Other	2 (40)	1 (20)	0 (0)
**Diagnosis**				
	Heart failure/cardiomyopathy	1 (20)	4 (80)	2 (67)
	Other cardiovascular disease	4 (80)	1 (20)	1 (33)

Similarly, although all participants had access to the internet at home, 54% (7/13) spent less than 5 hours per week on the internet, whereas 46% (6/13) spent more than 5 hours per week on the internet at home. Nevertheless, the majority also reported at least being somewhat comfortable with using online forums or message boards (62%, 8/13); and 5 participants (38%, 5/13) regularly used online forums or message boards for personal use (Table 2).

### Overall Satisfaction and General Comments

#### Satisfaction With the e-Forum

Evaluation of the user satisfaction assessment indicated that, on average, participants in all 3 rounds were satisfied with their experience in using the e-Forum (Table 3). Similarly, the majority of participants made at least one comment regarding their overall satisfaction with the e-Forum, and the number of satisfactory comments per participant increased from 3.5 in round 1 to 5 in round 3. Unique comments included general statements of satisfaction, expressions of satisfaction with the moderated aspects of the forum, as well as satisfaction with the opportunity to connect with other patients with similar conditions (Tables 4 and 5).

#### Description of Use

Participants from all 3 rounds made a total of 41 individual comments describing how they would use the e-Forum. Participants indicated that they would use the e-Forum to exchange advice regarding lifestyle behavior change and to share/gather information regarding the management of their cardiac condition. They also indicated that they might enlist the help of family members when using the e-Forum, and that this interface may also be used to provide additional support for family members of cardiac patients. Participants said that other cardiac patients would also likely be interested in using the e-Forum for additional support and resources (Tables 4 and 5).

#### Potential Barriers

Participants in all rounds also speculated that there might be potential barriers to accessing or using the e-Forum for other cardiac patients. There was a decrease in the total number of comments made regarding potential barriers between round 1 (12 comments) and round 3 (4 comments). Potential barriers included lack of access to the internet, poor computer skills, and self-consciousness about typing or general ability to use computers. Other barriers included the potential unwillingness of some cardiac patients to share their experiences in managing their condition (Tables 4 and 5).

**Table 2:** Self-reported computer and internet use.

Computer and internet usage variables		Round 1 (n=5), n (%)	Round 2 (n=5), n (%)	Round 3 (n=3), n (%)
**Use of computer: work**				
	Yes	3 (60)	2 (40)	2 (67)
	No	0 (0)	0 (0)	1 (33)
	Not applicable	2 (40)	3 (60)	0 (0)
**Use of internet: work**				
	Yes	3 (60)	2 (40)	3 (100)
	Not applicable	2 (40)	3 (60)	0 (0)
**Use of computer: home**				
	Yes	5 (100)	5 (100)	2 (67)
	No	0 (0)	0 (0)	1 (33)
**Hours spent on computer: home**				
	<5 hours per week	4 (80)	1 (20)	1 (33)
	>5 hours per week	1 (20)	4 (80)	1 (33)
	Not applicable	0 (0)	0 (0)	1 (33)
**Use of internet: home**				
	Yes	5 (100)	5 (100)	3 (100)
**Hours spent on internet: home**				
	<5 hours per week	3 (60)	2 (40)	2 (67)
	> 5 hours per week	2 (40)	3 (60)	1 (33)
**Use of online forums/message boards for personal use**				
	Yes	1 (20)	2 (40)	2 (67)
	No	4 (80)	3 (60)	1 (33)
**Level of comfort: computers**				
	Somewhat or comfortable	4 (80)	3 (60)	1 (33)
	Very comfortable	1 (20)	2 (40)	2 (67)
**Level of comfort: internet**				
	Comfortable	4 (80)	3 (60)	1 (33)
	Very comfortable	1 (20)	2 (40)	2 (67)
**Level of comfort: online forums/message boards**				
	Not at all comfortable	1 (20)	0 (0)	0 (0)
	Somewhat or comfortable	3 (60)	3 (60)	1 (33)
	Very comfortable	0 (0)	0 (0)	1 (33)
	Not sure	1 (20)	2 (40)	1 (33)

### Navigation: Task Navigation and Comments

#### Task Navigation

All participants were able to successfully navigate the e-Forum, with correct navigations per participant increasing from 13.8 in round 1 to 14.7 in round 3. Successful navigation included the ability to complete the specific steps to use the various features of the forum (eg, logging on, playing the tutorial video, editing profiles, and submitting and managing posts). Each participant also made at least one navigation error during the course of the study session. Nevertheless, the average number of navigation errors per participant decreased across the 3 rounds (5 in round 1 to 3.7 in round 3). Common navigation errors included difficulty finding the “edit profile,” “rate this post,” and “bookmark” buttons because of button placement or poor labeling (Table 4). Common navigation errors were addressed in changes made to the e-Forum between each round. See below for details.

**Table 3:** User satisfaction with e-Forum.

User satisfaction assessment	Round 1 (n=5), mean (SD)	Round 2 (n=5), mean (SD)	Round 3 (n=3), mean (SD)
I learned how to use the forum quickly and easily	4 (0.6)	5 (0.6)	5 (0.6)
I can find the information I am looking for on the forum with no problems	4 (1.3)	5 (0.6)	5 (0.6)
I can make and reply to posts on the forum with no problems	4 (0.8)	4 (0.9)	5 (0.6)
I am confident that I can remember how to get around the forum on my own every time I log on	4 (0.6)	4 (0.6)	4 (1.0)
If I get lost on the forum, I am confident that I can find my way again	4 (0.6)	4 (0.8)	4 (1.0)
I am satisfied with the forum	4 (1.1)	4 (0.0)	4 (0.6)
I would use the forum regularly to help me better manage my heart condition	5 (0.5)	4 (1.1)	4 (0.6)

**Table 4:** Content analysis of usability of e-Forum.

Themes		Round 1 (n=5)				Round 2 (n=5)				Round 3 (n=3)			
		C/I^a^, n	Ucs^b^, n	P^c^, n	Mean # of C/I per P^d^	C/I, n	Ucs, n	P, n	Mean # of C/I per P	C/I, n	Ucs, n	P, n	Mean # of C/I per P
**Content**													
	Positive comments	14	5	4	3.5	10	3	5	2.0	8	2	3	2.7
	Negative comments	8	5	3	2.7	16	9	4	4.0	4	3	2	2.0
	Neutral comments	19	12	5	3.8	20	12	4	5.0	14	5	3	4.7
**Navigation**													
	Positive comments	12	2	4	3.0	9	2	5	1.8	5	2	2	2.5
	Negative comments	1	1	1	1.0	4	2	3	1.3	1	1	1	1.0
**Layout**													
	Positive comments	15	5	5	3.0	21	5	5	4.2	10	4	3	3.3
	Negative comments	10	6	2	5.0	15	8	4	3.8	1	1	1	1.0
**Task navigation**													
	Correct navigation	69	17	5	13.8	70	16	5	14.0	44	16	3	14.7
	Navigation errors	21	13	5	4.2	19	9	5	3.8	11	7	3	3.7
**General feedback**													
	Satisfaction with forum	14	3	4	3.5	9	3	5	1.8	15	3	3	5.0
	Potential barriers	12	4	4	3.0	5	2	3	1.7	4	1	2	2.0
	Description of use	18	5	5	3.6	15	4	5	3.0	8	3	3	2.7

^a^Comments or incidents.

^b^Unique comments.

^c^Participants reported.

^d^Comments or incidents per participant.

#### Positive Navigation Comments

The majority of participants (85%,11/13) gave positive feedback (26 total positive comments) with regard to their ability to navigate the e-Forum. Positive comments included expressions of overall ease of navigation and indications that participants found the e-Forum easier to navigate or to understand as they used it (Tables 4 and 5).

#### Negative Navigation Comments

At least one participant in all 3 rounds provided a minimum of one negative comment on their ability to navigate the e-Forum. A total of 6 negative navigation comments were made, including overall difficulty with navigation and indications that the e-Forum was too complex to navigate (Tables 4 and 5). Nevertheless, all participants were able to successfully complete study tasks with little or no assistance.

**Table 5:** Sample comments of each of the themes.

Themes		Examples
**Content**		
	Positive comments	“...the [highlighted] topics...active living, eating healthy, get motivated,...I feel these are all the topics that...people would be interested in.” [B5, round 2]
		“...I’m...assuming that with every reply, I’ll get something in my inbox as well, so that’s...good...[because]...after its been forwarded it’ll just send me an email and then I’ll know okay, someone’s replied and...have the answer to my question okay...that’s good.” [B4, round 2]
	Negative comments	“I would definitely use [the forum] if there was more...information and the content was more rich.” [A5, round 1]
		“...it wasn’t clear to me what criteria I was supposed to use [to rate posts]...[there] was a bit of guess work involved in there.” [A2, round 1]
	Neutral comments	“...I would make it mobile friendly, I don’t know if it’s a mobile friendly site.” [ B5, round 2]
		“Another [topic] is sleeping...Sleeping is critically important...I would find it really interesting to understand how people approach that...what they think are good rules to follow, how they're doing it...” [C1, round 3]
**Navigation**		
	Positive comments	“It’s very clear...I think once you go through it once or twice, it [is] very simple to...follow.” [A4, round 1]
		“This is very user friendly; I don’t think navigating it is a problem...It’s fairly intuitive and easy to follow. I don’t think anyone who uses the internet regularly should have any difficulty...navigating it.” [A5, round 1]
	Negative comments	“It was a little difficult [to navigate]...I should say not very difficult because I’ve had an idea with the keyboard and I've looked at [something] similar to this...but [for] some people it may be very intimidating for them.” [B3, round 2]
		“...there’s too many pop up boxes...there’s too many steps...[for something that] could be 1 step there’s...3 steps instead...and I feel like people will get confused...” [B5, round 2]
**Layout**		
	Positive comments	“I think [the layout is] very simple and nice; I like the simplicity, I like the use friendliness.” [A5, round 1]
		“It’s really well done, like the font and the color, and when you need to know something, it pops up where it needs to be...all of the buttons are great, when you touch them they work the first time...I think it’s an excellent website.” [C2, round 3]
	Negative comments	“[I liked least] the cumbersome aspects of the webpage, having to click on that edit button which I didn't know was an edit button in the first place, unless I roll my mouse over it...” [B1, round 2]
		“...[the layout is] a little too busy; keep it simple is the right idea.” [ A1, round 1]
**General feedback**		
	Satisfaction with forum	“The internet is a source of a lot of useful information but can also be a source of a lot of...misinformation, so...the moderation must be there, otherwise you [end up] working against your own best interests.” [A2, round 1]
		“I think [this forum]...would come in handy [for other heart patients] ... [to] check on how they’re doing, and how other people are doing. And it’s pretty easy to use it on a computer.” [C3, round 3]
	Potential barriers	“When it comes to personal life...like health... [people] are not as open, for whatever reason...sometimes they don’t want to talk about it, they just want to leave it alone.” [ A3, round 1]
		“There [are] some people that…wouldn’t use it at all, just [because] they don’t have a computer [or] maybe they’re not going to adapt to a computer program and find it very difficult [and] intimidating.” [A4, round 1]
	Description of use	“Yeah [I would use a forum like this]. I would go in and see once you start having situations...with your health you see what other people are doing for exercising, eating healthy…” [A3, round 1]
		“It’s the time in between your [appointments] when you have all the questions [about your diagnosis or procedure]...so having something as a resource to refer to would be something good.” [B4, round 1]

### Content

#### Positive Content Comments

A majority of participants (92%, 12/13) provided positive feedback regarding the content presented in the e-Forum. Participants indicated that they were satisfied with features/functionality of the e-Forum (eg, appreciation of confirmation messages after submissions, spell-checking, bookmarking, or tool tips) as well as the sample information provided (eg, indication that video and highlighted topics were helpful; Tables 4 and 5).

#### Negative Content Comments

Negative comments regarding the content of the e-Forum were made in each round, with a total of 9 participants making 28 such comments throughout the course of the study. Negative content feedback included dissatisfaction with certain features or functionalities (eg, unclear rating criteria, tutorial video being overwhelming, and lack of spell-checking feature) and with the sample information provided in the e-Forum (eg, finding certain highlighted topics not relevant to their experience or that content was not comprehensive enough; Tables 4 and 5).

#### Neutral Content Comments

All participants made at least one neutral comment about the content of the e-Forum. Neutral comments included suggestions for additional features or functionalities of the e-Forum (eg, suggestions to create a search button or to host live support groups), suggestions for information to be provided on the e-Forum (eg, suggestions for additional highlighted topics or videos), and suggestions to create different forum groups based on varying health status (eg, diagnoses or lifestyles; Tables 4 and 5).

### Layout

#### Positive Layout Comments

Every participant made at least one positive comment regarding the layout of the e-Forum. In total, 46 positive layout comments were made. Positive comments included satisfaction with buttons (eg, appropriate size and color), with font size (eg, easy to read or see), with colors (eg, attractive), and with the overall layout of the forum (eg, simple and easy to use; Tables 4 and 5).

#### Negative Layout Comments

At least one participant from each round expressed a negative comment regarding the layout of the e-Forum. However, such comments decreased from 5 to 1 comment per participant from round 1 to round 3. Negative comments included expressions of dissatisfaction with overall layout of the e-Forum (eg, layout too complex), font size (eg, too small or inconsistent), colors (eg, inconsistent or dated), or buttons (eg, not clearly labeled; Tables 4 and 5).

### Changes Made to Forum

#### From Round 1 to Round 2

On the basis of the feedback provided by participants in round 1, various changes were made to the design of the e-Forum to improve usability for the subsequent round of participants. For example, buttons were moved and/or renamed to enhance visibility and accessibility. Text boxes were reformatted, and tool tips and button labels were changed or added (eg, changing “Return to Forum” to read “Go Back”) throughout the forum to improve the accessibility of associated features or functions. Finally, suggested changes were made to the layout of the forum, including changes in background and font color.

#### From Round 2 to Round 3

On the basis of the feedback provided by participants in round 2, more changes were made to the e-Forum to improve usability. Buttons were moved and/or renamed to enhance visibility and accessibility, and they were reformatted to improve consistency in layout. A keyword search feature was added to the e-Forum. Grammar and spell-checking features were added to textboxes; contact information for the research team, including expected response times, was also added to the e-Forum. See Figures 1-3 for a sample of the final version of the e-Forum at the completion of this usability study.

## Discussion

### Principal Findings

The e-Forum was designed to facilitate the establishment of a reliable and accessible online community for cardiac patients. This usability study was conducted to ensure that our e-Forum was user-friendly and accessible to our target patient population. An iterative design was used such that after each round of study sessions, changes were made to the e-Forum in response to participant feedback. Feedback included general reflections of user experiences as well as positive, negative, and neutral comments on the content, navigation, and layout of the e-Forum.

Overall, participants across all 3 rounds were highly satisfied with the e-Forum. Between rounds 1 and 3, expressions of satisfaction with the e-Forum increased, and fewer potential barriers were reported. Participants indicated that it would be helpful to speak with other cardiac patients and that they were particularly satisfied with the moderated aspect of the e-Forum. Participants indicated that they would use the e-Forum to exchange lifestyle behavior advice and general information regarding their health management with other patients. Having the moderation feature reassured them that the information they obtained would be reliable and safe. They also predicted that their family members would likely use the e-Forum on their own or together with the patients to obtain information and support.

As improvements were made to the e-Forum based on participant feedback, positive comments related to layout increased from the initial to the final round, whereas negative layout comments decreased. Moreover, negative comments related to content and the number of navigation errors decreased between rounds 1 and 3. These outcomes indicated that modifications made to the layout (eg, changes in colors and font sizes), as well as the content (eg, changes in descriptors and features, including the addition of the keyword search) of the e-Forum, likely improved the overall user experience and ease of use when interacting with our online community.

### Limitations

The results of this study indicate that our e-Forum would likely be accessible to a diverse array of cardiac patients. However, there are some limitations to consider as efforts are made to disseminate this e-Forum to the wider patient population. For example, although the age of participants in this study was well representative of the target user population (10 participants were aged older than 50 years), those who agreed to participate in this study were primarily white, with at least some postsecondary education, and with self-reported experience and comfort using computers and the internet.

It is possible that our findings may be limited in generalizability, as the overall population of cardiac patients is more culturally and educationally diverse. Nevertheless, feedback provided by participants in this study also suggested that individuals from diverse backgrounds may actually be *more* comfortable asking questions about lifestyle behaviors on our e-Forum. These comments are congruent with other studies that have found that users from rare or geographically dispersed backgrounds may be more likely to feel confident in exchanging experiences and advice with regard to their health management within an online community [33]. Similarly, although some participants suggested that the wider patient population may be less comfortable with or have limited access to the internet, such concerns may not be relevant, as it has been established that the majority of individuals in North America have access to the internet [34,35].

### Future Directions

Given the limitations of this study, future studies may work to recruit a more diverse sample of patients to ensure ease of use of the e-Forum across a wide range of patient demographics and experiences. Future studies may also use back-end analytics to assess how participants organically use the e-Forum (eg, how often, for how long, and which features they use most frequently) to gather additional information about how best to maximize the usability of the e-Forum. From a design perspective, future versions of the e-Forum may also increase usability by programming additional features, including making the e-Forum more mobile-friendly, including of speech recognition software, creating additional tutorial videos, allowing users to change font sizes, and offering the e-Forum in multiple languages. Moreover, it will be important to assess the e-Forum’s ability to ultimately promote continued user engagement in Web-based lifestyle counseling programs and long-term self-care adherence.

### Conclusions

In this study, we found evidence to support the usability of our newly designed e-Forum. After each study round, changes were made to the e-Forum based on user feedback. For example, buttons were moved and/or renamed to enhance visibility and accessibility and features, including but not limited to, a keyword search, and tool tips were added throughout the e-Forum. As a result, a diverse sample of cardiac patients, in terms of age and self-reported comfort with computers/internet, were able to successfully navigate the e-Forum. Moreover, these users indicated satisfaction with the layout and content of the e-Forum and expressed interest in using this tool for practical and emotional support in managing their CVD. The high user satisfaction ratings indicate that the e-Forum provided an acceptable user experience. In sum, these findings support this tool and its potential role in promoting long-term lifestyle behavior change when paired with existing e-counseling programs, such as the CHF-CePPORT program [10].

## Appendix

Multimedia Appendix 1 Task instructions.

## Abbreviations

C/I: comments or incidents
CHF-CePPORT: Canadian e-Platform to Promote Behavioral Self-Management in Chronic Heart Failure
CVD: cardiovascular disease
HF: heart failure
P: participant
Ucs: unique comments
