# Supplementary material for: A Moderated e-Forum for Adults With Cardiovascular Disease: Usability Study
Source: JMIR Hum Factors. 2018 May 18;5(2):e20. doi: 10.2196/humanfactors.8820 (PMC5984275; doi:10.2196/humanfactors.8820)
Supplement: Multimedia Appendix 1 [file humanfactors_v5i2e20_app1.pdf]

Appendix A

Task Instructions	
**A**	1. **Log onto our website.**<br>Username:__________________________________<br>Password:__________________________________<br>2. **Click into "My e-Community"**<br>*3.* **Watch the tutorial video.**
**B**	1. **Enter the "e-forum."**<br>2. **Go to "My Profile."**<br>3. **Enter following profile description:**<br>*I joined this forum to connect with other patients diagnosed with heart failure and to gain additional support from providers who have experience with patients like me.*<br>4. **Submit this profile.**
**C**	1. **Open the most recently submitted post.**<br>2. **Give this post a rating of "3 stars."**<br>3. **Bookmark this conversation and enter the following description before saving.**<br>Description: *Recipes*<br>4. **Reply to this post with the following information.**<br>*Thanks for this recipe, it was delicious! Anyone have suggestions for breakfast foods that are healthy and tasty?*
**D**	*1.* **Enter the "Topics" page of the e-forum.**<br>*2.* **View the posts listed under "Getting Motivated."**<br>*3.* **Add a new post and enter the following information.**<br>Subject: *Exercising when Stressed*<br>Text: *It's hard for me to stay motivated when I'm stressed. Does anybody else experience this too? Or am I the only one?*
